# DNA cytosine deamination is associated with recurrent Somatic Copy Number Alterations in stomach adenocarcinoma

**DOI:** 10.3389/fgene.2023.1231415

**Published:** 2023-10-06

**Authors:** Yilin Shi, Huangxuan Shen

**Affiliations:** ^1^ The College of Letters & Science, University of Wisconsin–Madison, Madison, WI, United States; ^2^ State Key Laboratory of Ophthalmology, Zhongshan Ophthalmic Center, Sun Yat-sen University, Guangzhou, China

**Keywords:** stomach adenocarcinoma, DNA cytosine deamination, hotspots, Somatic Copy Number Alterations, CCLE database, TCGA database

## Abstract

Stomach Adenocarcinoma (STAD) is a leading cause of death worldwide. Somatic Copy Number Alterations (SCNAs), which result in Homologous recombination (HR) deficiency in double-strand break repair, are associated with the progression of STAD. However, the landscape of frequent breakpoints of SCNAs (hotspots) and their functional impacts remain poorly understood. In this study, we aimed to explore the frequency and impact of these hotspots in 332 STAD patients and 1,043 cancer cells using data from the Cancer Genome Atlas (TCGA) and Cancer Cell Line Encyclopedia (CCLE). We studied the rates of DSB (Double-Strand Breaks) loci in STAD patients by employing the Non-Homogeneous Poisson Distribution (λ), based on which we identified 145 DSB-hotspots with genes affected. We further verified DNA cytosine deamination as a critical process underlying the burden of DSB in STAD. Finally, we illustrated the clinical impact of the significant biological processes. Our findings highlighted the relationship between DNA cytosine deamination and SCNA in cancer was associated with recurrent Somatic Copy Number Alterations in STAD.

## Introduction

STAD is ranked as the fifth most commonly diagnosed cancer in 2020 and is the third leading cause of cancer-related deaths worldwide (Zhou et al., 2020). China has the highest incidence rate of STAD, accounting for 49.9% of global cases, with approximately 498,000 deaths in 2019 (Zhu et al., 2019). In general, external mutagens, such as smoking, and alterations of certain biological functions, such as deficiencies in DSB repair mechanism and *APOBEC* enzymatic activities, would result in specific mutational signatures (Guo et al., 2021). Recent studies also highlighted the involvement of Microsatellite Instability, Tumor Mutation Burden, and SCNAs in the progression of Gastric Cancer (Chen et al., 2022). Filaggrin (*FLG*) mutation led to increased gastric cancer sensitivity to 24 chemotherapeutic drugs, suggesting a potential protective factor (Yicheng et al., 2022). GLP2R knockdown was shown to significantly inhibit the proliferation and migration of gastric cancer cells *in virto* (Fu et al., 2022). Despite extensive research in STAD, the underlying pathogenesis and etiology of the disease remained elusive. Mutagenic processes only partially accounted for observed mutational signatures in cancer patients (Watkins et al., 2020). Therefore, further investigation into frequent SCNAs in STADs was crucial.

SCNAs resulted in multiple copy gains or losses of specific DNA fragments on homologous chromosomes (Hovhannisyan et al., 2019). They arose from inter-related processes of replication stress, spindle multipolarity, mitotic errors, and breakage–fusion–bridge cycles (Steele et al., 2022). Genome doubling and ongoing dynamic chromosomal instability resulted in the evolution of driver SCNAs (Jamal-Hanjani et al., 2017). HR, an evolutionary-conserved mechanism, playing a role in a subtle balance between genome stability and diversity, was a DNA repair pathway (Carr and Lambert, 2013). HR deficiency underlying the DSB-repair mechanism has been identified as a major cause of SCNAs in cancer cells (Hastings et al., 2009). Recent Studies demonstrated a strong correlation between large Somatic Copy Number Alterations (SCNAs) and the development of developmental disorders and cancer (Macé et al., 2018). In particular, SCNAs in STAD have been found to exhibit various signatures involving cancer-related genes such as *TP53, PIK3CA*, and *ARID1A*. These signatures include Diploid with zero whole-genome doublings, chromothripsis amplification, and loss of heterozygosity. The drivers of some signatures of SCNAs in STAD have been identified as *MDM2, EGFR, CCNE1, MYC, and ERBB2* (Hovhannisyan et al., 2019). A computational method applied to characterize aneuploidy in samples of tumors according to coordinated aberrations in the expression of genes in each chromosomal region has been developed (Carter et al., 2006). However, the specific genes and their functions within SCNA hotspots remain unknown. Therefore, it is crucial to identify relevant genes within hotspots of SCNAs to investigate their functions.

**FIGURE 1:**
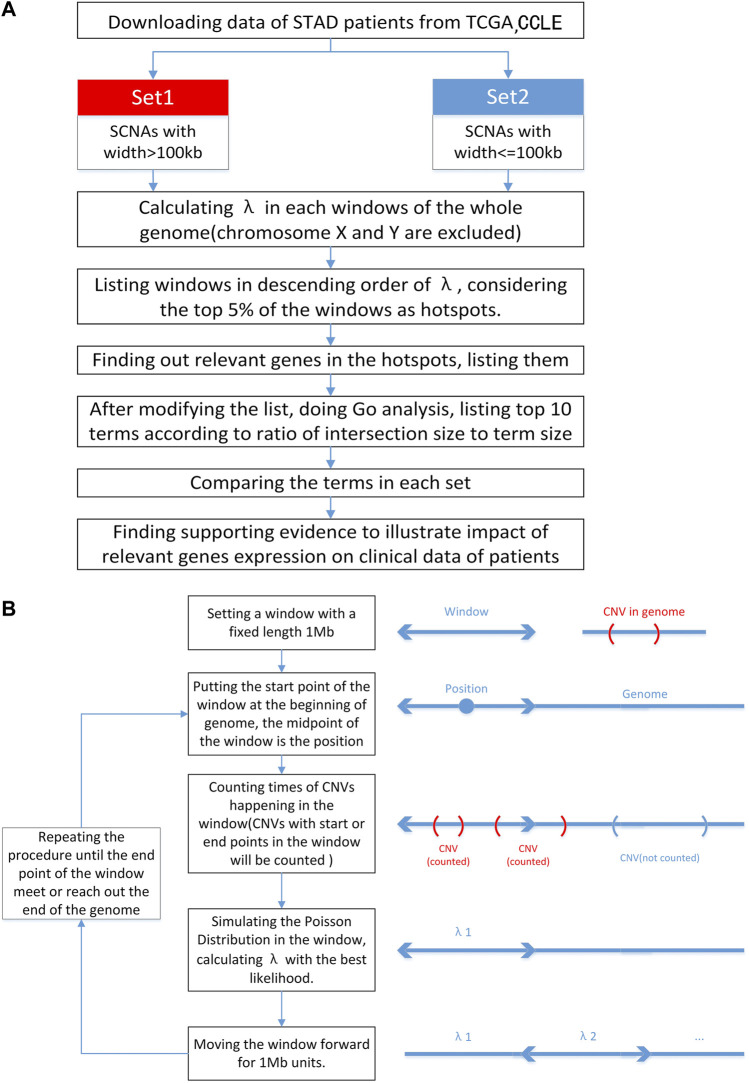
Flowcharts of the procedure. **(A)** The schematic view of this study. In this study, we classified SCNAs in terms of width. Excluding chromosome X and Y, we divided genome into parts and simulated the Poisson Distribution to calculate λ in each window to find out hotspots. Then relevant genes were found, GO analysis was done after modification of gene list and top 10 terms were listed. At last relationship between some relevant genes and SCNA burden was illustrated by T-test and survival analysis was made. **(B)** Flowchart of calculating λ in different positions of genome. We set a window on genome and calculate the λ in this window. The calculation lasts until the end of the genome.

Here, we analyzed the distribution of the DSB points of 179166 SNCA events across 332 STADs from TCGA and 872216 SCNA events across 1,043 cancer cells from CCLE. We identified 145 hotspots of recurrent DSB in STAD and found that DNA Cytosine Deamination was associated with the load of SCNAs in STADs and the corresponding clinical outcome. The schematic view of this study and methods of calculating λ were provided (Figures 1A, B).

## Material and methods

### Raw data

The data on SCNAs in STAD patients, overall survival of STAD patients, gene expression in STAD patients, and locations of genes were obtained from TCGA (https://tcga-data-secure.nci.nih.gov/tcgafiles/tcga4yeo/tumor/prad/cgcc/broad.mit.edu/genome_wide_snp_6/snp/). The data was available in appendices. The version of the genome was hg19 (https://genome.ucsc.edu/cgi-bin/hgTracks?db=hg19&lastVirtModeType=default&lastVirtModeExtraState=&virtModeType=default&virtMode=0&nonVirtPosition=&position=chr2%3A25383722%2D25391559&hgsid=1653852260_7skZ6eFcyA94KTIXSNz9IEKYP60J). The data of SCNAs in STAD cell lines and expressions of relevant genes were obtained from CCLE (https://depmap.org/portal/download/all/). The two sets of data were analyzed separately.

### Classification of SCNAs

The data was imported into RStudio 4.2.2 and classified into two sets: large-scale SCNAs with a width >100 kb and SCNAs with a width≤100 kb (McCarroll et al., 2008). They were analyzed separately to better understand their characteristics and implications. For ease of reference, SCNAs with a width >100 kb and SCNAs with a width≤100 kb were called Set1 and Set2, respectively (Table 1). See supporting material.

**TABLE 1:** Convenient code for two sets of experiments. Convenient code for two sets of experiments.

Set of experiment	Procedure of experiment
Set1	Analyzing SCNAs with width> 100 kb
Set2	Analyzing SCNAs with width =< 100 kb

### Calculation of λ

We hypothesized that the rate of DSB follows a Poisson Distribution with the parameter λ. To obtain λ based on the number of DSB events in each locus, a log-likelihood function was utilized to estimate λ values, taking into account the deviation from a perfect Poisson Distribution observed in the data (van Opheusden et al., 2020).

The study analyzed two datasets to determine the burden of SCNAs in specific genomic regions. The SCNA burden, defined as the number of SCNAs, was calculated for each patient within 1 Mb windows along the chromosomes.

To determine whether an SCNA fell within a window, the following criteria were applied: if the start point of the SCNA was smaller than the endpoint of the window, and the endpoint was larger than the start point of the window, then the SCNA was counted within the window.

Using RStudio, λ was estimated for each window. The parameter λ, representing the average occurrence of SCNAs within a window, was calculated using log-likelihood functions. It was observed that the distribution of λ within the windows did not perfectly match the Standard Poisson Distribution models.

To visually represent the distribution of λ, histograms of log2(λ) were plotted. Additionally, Manhattan plots were generated, using a threshold value approximately equal to the minimum value of λ observed in the hotspots, to illustrate the hotspots.

### Identification of hotspots

Hotspots were determined based on the top 5% of the λ values, as this threshold was considered statistically significant. The information regarding the hotspots in both sets of experiments can be found in the appendices, which include details such as the location of the hotspot and the corresponding λ value. We also identified the relevant genes located within these hotspots.

### Gene lists modification

Before doing the GO analysis, hyper-polymorphic genes such as genes in *OR, TAS, IGH, IGK, IGL, HLA* family, and genes in the list of *HLA* (See appendix) were excluded from the analysis. Odor genes and taste receptor (TAS) (Keller et al., 2007; Carrai et al., 2017), HLA molecules (Dendrou et al., 2018), IGK rearrangements (Jackson et al., 2012), IgH class switching (Stavnezer and Schrader, 2014), Immunoglobulin light (IgL) chains (Edholm et al., 2011) were proven to be highly variable in all individuals. Their variability could potentially interfere with the GO analysis. By excluding these genes, we aimed to focus on identifying less variable but significant genes associated with the progression of stomach adenocarcinoma (STAD) and determining corresponding significant functional terms.

### Enrichment analysis of differentially expressed genes

After modification, GO analysis was performed on GProfiler (Raudvere et al., 2019). The website was available at: https://biit.cs.ut.ee/gprofiler/gost. We tested the statistical enrichment of expression genes related to hotspots in the KEGG path. The ratio of intersection size to term size was calculated. Terms with the top 10 ratio value were selected to be graphed in a bubble graph. The adjusted *p*-value of all terms was smaller than 0.05, which illustrated that the terms were significantly enriched by expressed genes.

### Statistical analysis

Violin plots visualized the relationships between the expression of significant genes identified in GO analysis and SCNA burden. The patients were divided into two groups, namely, “High” and “Low”, based on their median SCNA burdens. Both consisted of an equal number of patients. In addition, T-tests demonstrated the relationship between the APOBEC family copy number statuses and SCNA burden. If the start point of the SCNA was smaller than the endpoint of the analyzed *APOBEC* gene, and the endpoint of the SCNA exceeded the start of the *APOBEC* gene, patients with this SCNA were classified as “Mutant”, while the other patients were classified as ‘Wild’. T-tests with *p*-values less than 0.05 were considered statistically significant, and corresponding genes were deemed significant.

### Survival analysis

To explore the relationship between SCNA burden and overall survival, we utilized the R packages survival (version 3.4-0) and survminer (version 0.4.9) to generate Kaplan-Meier curves. Furthermore, patients were categorized based on the expression of *APOBEC* genes for survival analysis. Each survival analysis comprised three groups: Low, Median, and High. Results with *p*-values smaller than 0.05 were deemed statistically significant.

## Result

### Calculation of λ

**FIGURE 2:**
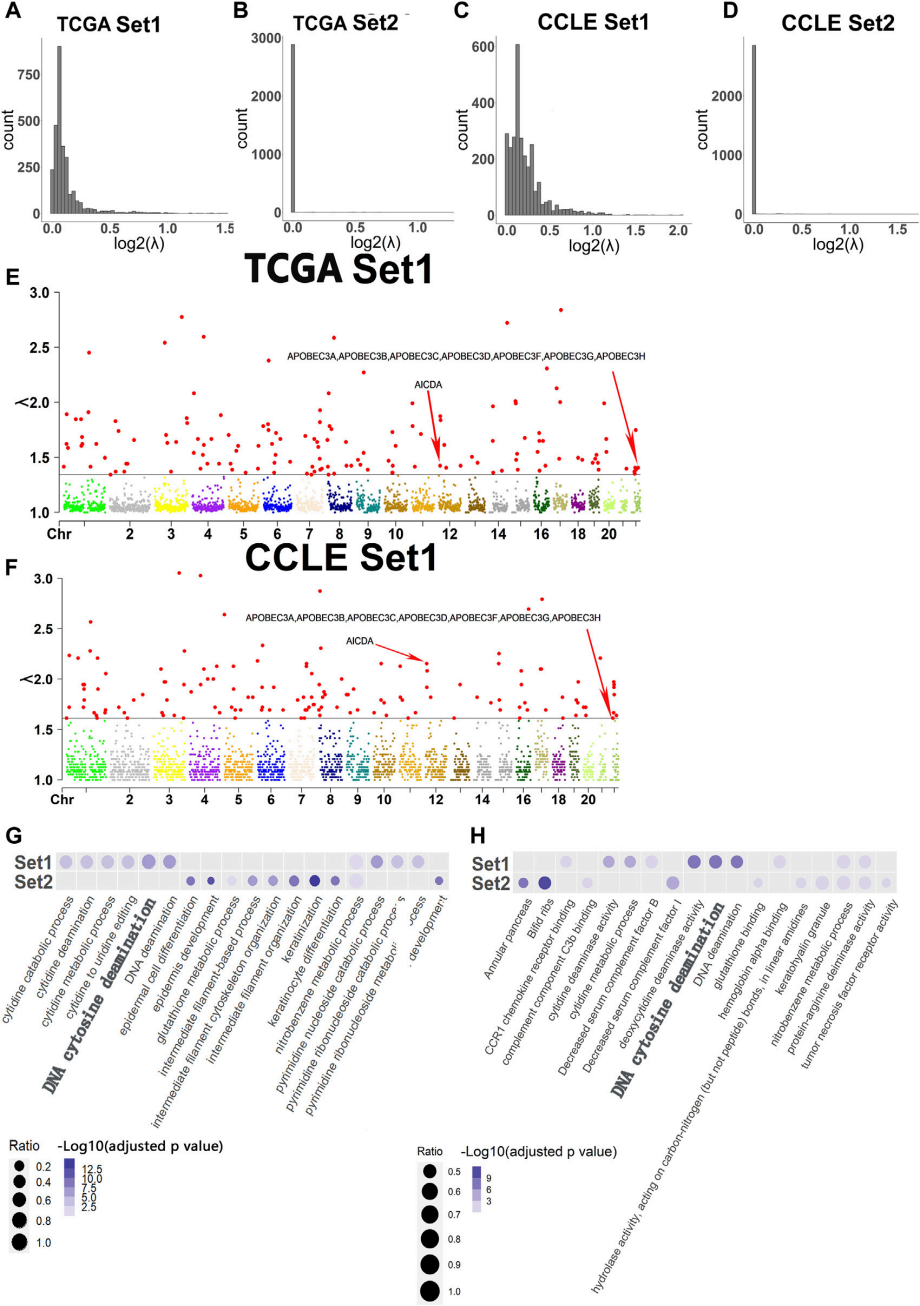
Histogram of log2(λ), Manhattan plot of λ in Set1 and Top 10 terms of GO analysis in two sets of experiments. **(A)** Histogram of log2(λ) in Set1 for TCGA data set. **(B)** Histogram of log2(λ) in Set2 for TCGA data set. **(C)** Histogram of log2(λ) in Set1 for CCLE data set. **(D)** Histogram of log2(λ) in Set2 for CCLE data set. **(E)** Manhattan plot of λ in Set1 for TCGA data set. The red points which are larger than other colorful points below are λ values that are considered as hotspots in each chromosome. The corresponding hotspots containing *APOBEC* genes have a relative low value of λ than other λ of hotspots. The red arrows represent the values of λ of hotspots which contain genes relative to DNA cytosine deamination found in GO analysis. **(F)** Manhattan plot of λ in Set1 for TCGA data set. The red points which are larger than other colorful points below are λ values that are considered as hotspots in each chromosome. The corresponding hotspots containing *APOBEC* genes have a relative low value of λ than other λ of hotspots. The red arrows represent the values of λ of hotspots which contain genes relative to DNA cytosine deamination found in GO analysis. **(G)** Top 10 terms of GO analysis in Set1 and Set2 for TCGA data set. **(H)** Top 10 terms of GO analysis in Set1 and Set2 for CCLE data set.

According to data from TCGA and CCLE, the majority of the log2(λ) values in Set1 (Width >100 kb) ranged from 0 to 0.5, while most of the log2(λ) values in Set2 (Width≤100 kb) were close to 0 (Figures 2A–D). Estimation revealed that the minimum value of λ in hotspots of TCGA Set1 was around 1.4, while the CCLE Set1 was around 1.6. In TCGA Set2 and CCLE Set2, the minimum value may be 1. Since the minimum value of Set2 was close to 1, which represents the overall minimum value of λ, the Manhattan plot of Set2 won’t be displayed. The Manhattan plot of Set1 with a threshold value of approximately 1.4 and 1.6, respectively, was plotted (Figures 2E, F).

### 145 hotspots and relevant genes

A total of 145 hotspots were identified from the analysis of 2,897 windows in Set1 and Set2. In TCGA Set1, 1,437 genes were found within these hotspots, and after modification, 1,230 genes remained. Similarly, in TCGA Set2, 452 genes were located within the hotspots, and 395 genes remained after modification. In CCLE-Set1, 1,462 genes were found within these hotspots, and after filtering for genes whose variability could potentially interfere with the GO analysis, 1,265 genes remained. In CCLE Set2, 1,458 genes were found within these hotspots, and 1,336 genes were left after the filtering (Table 2). The complete list of genes before and after modification for both sets of experiments can be found in the appendices, along with additional information on the hotspots.

**TABLE 2:** Minimum λ of hotspots, number of relevant genes before and after modification in two sets of experiments. Minimum λ of hotspots, number of relevant genes in two sets of experiments.

Experiment	Minimum λ of hotspots	Number of associated genes (before modification)	Number of associated genes (after modification)
Set1	1.342	1,437	1,230
Set2	1	1,337	1,277

### *APOBEC*-related DNA cytosine deamination was overrepresented in genes affected by DSBs

The GO analysis revealed that DNA Cytosine Deamination appeared among the top 10 terms in Set1. However, the top 10 terms in Set2 did not show any significant functions, leading to the exclusion of Set2 from further analysis. Interestingly, the GO analysis also highlighted the presence of genes belonging to the *APOBEC* family in both TCGA-Set1 and CCLE-Set1. These *APOBEC* genes were found to be associated with hotspots that exhibited a relatively low value of λ compared to other hotspots. The analysis revealed that these genes were predominantly located in hotspots with λ values around 1.4 and 1.6 in two sets of data, respectively (Figures 2G, H).

### Association between the SCNA burden and APOBEC family copy number statuses, expression of *APOBEC* gene

**FIGURE 3:**
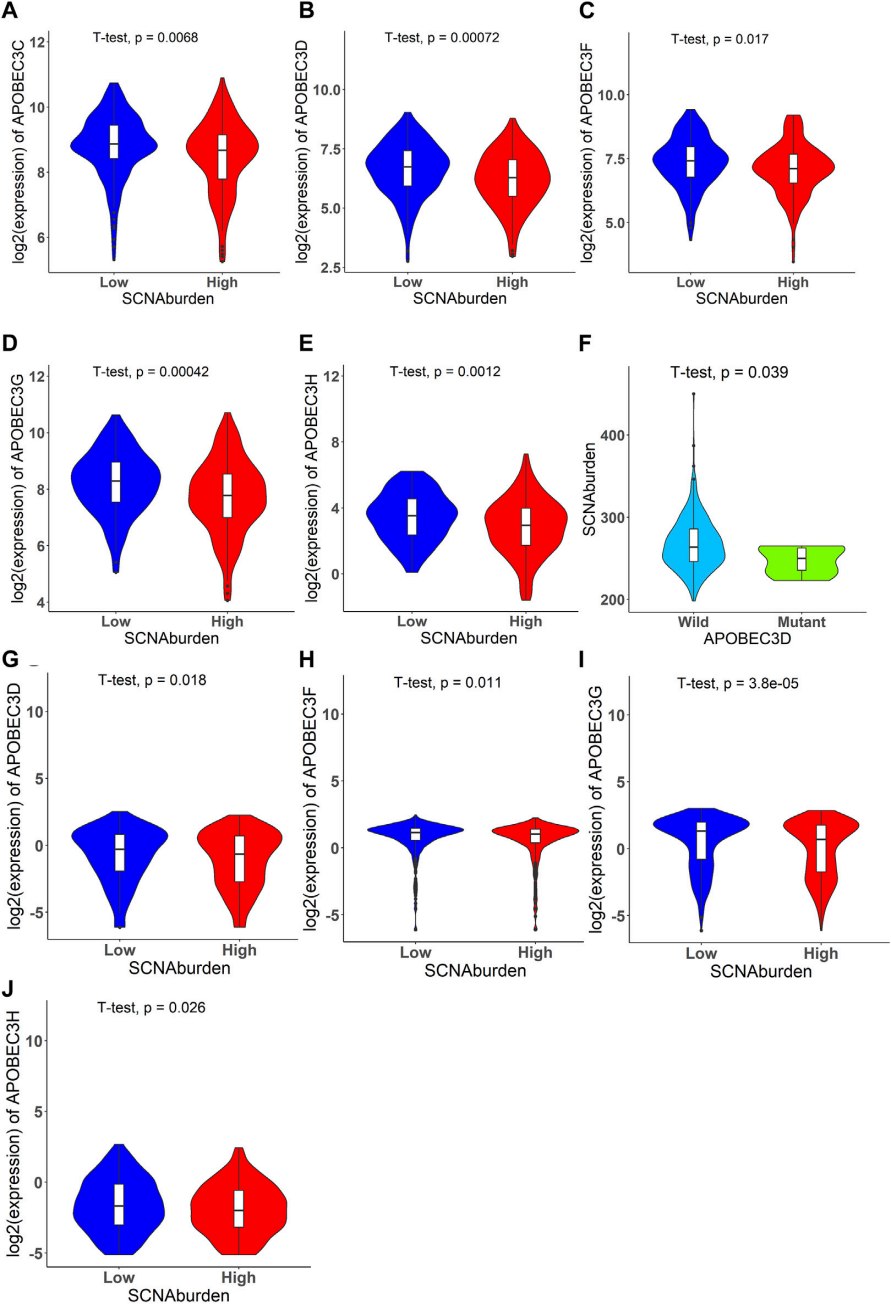
Significant violin plots that illustrate relationship between expression of *APOBEC* family genes and SCNA burden, DSB points affecting *APOBEC* genes and SCNA burden. The expression of *APOBEC3C, APOBEC3D, APOBEC3F, APOBEC3G, APOBEC3H* have relationship with SCNA burden. The DSB points affecting *APOBEC3D* have relationship with SCNA burden. **(A)** The violin plot of *APOBEC3C* for TCGA data set. **(B)** The violin plot of *APOBEC3D* for TCGA data set. **(C)** The violin plot of *APOBEC3F* for TCGA data set. **(D)** The violin plot of *APOBEC3G* for TCGA data set. **(E)** The violin plot of *APOBEC3H* for TCGA data set. **(F)** The violin plot of SCNA burden *versus* whether DSB points happened in *APOBEC3D* for TCGA data set. **(G)** The violin plot of *APOBEC3D* for CCLE data set. **(H)** The violin plot of *APOBEC3F* for CCLE data set. **(I)** The violin plot of *APOBEC3G* for CCLE data set. **(J)** The violin plot of *APOBEC3H* for CCLE data set.

In TCGA data, the results indicated a significant association between the expression levels of *APOBEC3C, APOBEC3D, APOBEC3F, APOBEC3G, APOBEC3H*, and SCNA burden (Figures 3A–E). The findings also revealed a significant association between DSB points affecting *APOBEC3D* and SCNA burdens (Figure 3F). In CCLE data, the results showed a significant association between the expression levels of *APOBEC3D, APOBEC3F, APOBEC3G, APOBEC3H,* and SCNA burdens (Figures 3G–J).

### Significance of SCNA burden and *APOBEC3C* expression on the prognosis of STAD patients

**FIGURE 4:**
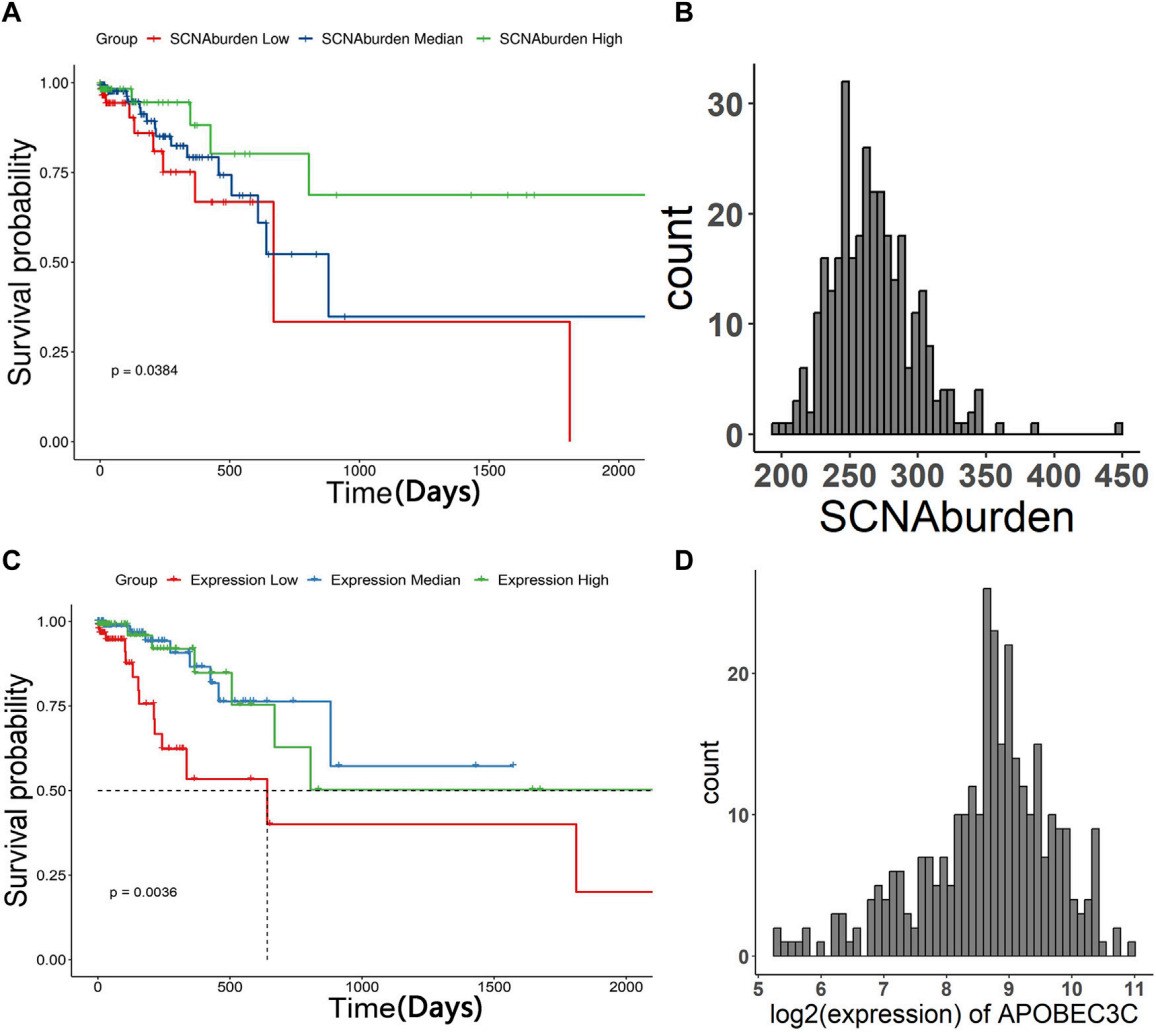
The Kaplan–Meier curve of patients of three groups and histogram of SCNA burden, histogram of log2 (expression of *APOBEC3C*). The patients were grouped in “SCNA burden low”, “SCNA burden median”, “SCNA burden high” and “Expression low”, “Expression median”, “Expression high” according to the expression of *APOBEC* genes, respectively. The result showed that patients with more SCNA tended to have better overall survival. Patients with median expression of *APOBEC3C* tended to have better overall survival. **(A)** The Kaplan–Meier curve of patients grouped in “SCNA burden low”, “SCNA burden median”, “SCNA burden high”. **(B)** Histogram of SCNA burden. **(C)** The Kaplan–Meier curve of patients grouped in “Expression low”, “Expression median”, “Expression high”. **(D)** Histogram of log2 (expression of *APOBEC3C*).

The cohort of 332 patients from TCGA was divided into three groups based on their SCNA burden. The survival analysis demonstrated that patients with higher SCNA burden tended to better overall survival compared to those with lower SCNA burden (Figures 4A, B). Additionally, we investigated the correlation between the expression of specific *APOBEC* genes and the overall survival of STAD patients. Similarly, the cohort of 332 patients was divided into three groups based on SCNA burden. The results indicated that patients with median expression of *APOBEC3C* tended to better overall survival, as opposed to those with high or low expression (Figures 4C, D).

## Discussion

The distribution of SCNAs has been extensively studied and found to be associated with various abnormal conditions, including autism spectrum disorder and Adrenocortical Carcinomas (Girirajan et al., 2013; Gupta et al., 2022). However, the relationship between the prognosis of STAD patients and hotspots of SCNAs remained unclear. Identifying genomic regions undergoing frequent alteration in human cancers was a powerful way to discover genes playing significant roles in oncogenesis (Beroukhim et al., 2010). By identifying genes located in the hotspots of SCNAs in STAD patients and understanding their functions, we may uncover new mechanisms of STAD development, novel cell signaling pathways, and targeted therapeutic approaches that can improve the precision of treatment for patients. It is worth noting that many SCNAs identified in one cancer type are present in multiple other cancer types (Beroukhim et al., 2010). In the case of *APOBEC* genes, it was proven that a common deletion in the *APOBEC3* gene was strongly associated with Breast Cancer risk (Long et al., 2013). Notably a signature attributed to the *APOBEC* family of cytidine deaminases was present in many cancer types (Alexandrov et al., 2013; Guo et al., 2018). Therefore, further research on SCNAs in STAD and *APOBEC* genes may yield valuable insights into the variations observed in other cancer types, ultimately advancing the overall treatment of various cancers. While the methods employed in this study may appear straightforward, there are still certain aspects that warrant discussion.

First, the result of GO analysis in Set1 indicated that genes related to DNA Cytosine Deamination may be highly variable in STAD patients. Previous research has demonstrated the beneficial role of DNA Cytosine Deamination in immunity and its detrimental effects on cancer (Shi et al., 2017). *APOBEC3*-mediated mutagenesis has been observed in various cancers (Butler and Banday, 2023). However, the specific mechanism underlying this association remains poorly understood. The DSB points that affect *APOBEC3D* appear to be linked to the SCNA burden, although the precise pattern of this relationship is still unknown. Future research should focus on unraveling the mechanisms through which DNA Cytosine Deamination impacts STAD patients.

Second, the findings of the study suggested a phenomenon that a higher burden of SCNAs in STAD patients was associated with improved clinical performance. This indicated that SCNAs may confer a survival advantage in STAD patients. Previous research showed that in the high tumor mutational burden group, nivolumab plus ipilimumab seemed to provide better clinical performance than nivolumab monotherapy (Hellmann et al., 2018). It was also shown that higher nonsynonymous mutation burden in tumors had a relationship with durable clinical benefit (Rizvi et al., 2015). However, the specific mechanism underlying the association requires further investigation. Interestingly, the study also observed that a median expression of *APOBEC3C* was associated with better clinical outcomes in STAD patients. This suggests that the expression of *APOBEC3C* may play a role in influencing the prognosis of STAD patients. Future research should focus on exploring the expression patterns of *APOBEC3C* and investigating its mechanism of action in affecting the prognosis of STAD patients.

Finally, the results of the analysis with TCGA data and CCLE data seemed to be the same. In the analysis for TCGA data, there was a significant association between the expression levels of *APOBEC3C, APOBEC3D, APOBEC3F, APOBEC3G, APOBEC3H,* and SCNA burden while the results showed a significant association between the expression levels of *APOBEC3D, APOBEC3F, APOBEC3G, APOBEC3H,* and SCNA burden with CCLE data. Both the result of GO Analysis for TCGA data and CCLE data revealed the significance of DNA Cytosine Deamination, which may suggest the endogeneity of DNA Cytosine Deamination. Future research should focus on the mechanism of DNA Cytosine Deamination.

## Conclusion

This research study identified a total of 145 hotspots associated with *APOBEC*-related DNA Cytosine Deamination in patients with STAD. These hotspots were found to be linked to recurrent SCNAs, suggesting a potential role in the progression of STAD. As a result, the *APOBEC* genes have emerged as potential targets for the treatment of STAD.

## Data Availability

The original contributions presented in the study are included in the article/Supplementary Material, further inquiries can be directed to the corresponding author.

## References

[B1] Alexandrov L. B. Nik-Zainal S. Wedge D. C. Aparicio S. A. J. R. Behjati S. Biankin A. V. (2013). Signatures of mutational processes in human cancer. Nature 500 (7463), 415–421. 10.1038/nature12477 23945592 PMC3776390

[B2] Beroukhim R. Mermel C. H. Porter D. Wei G. Raychaudhuri S. Donovan J. (2010). The landscape of somatic copy-number alteration across human cancers. Nature 463 (7283), 899–905. 10.1038/nature08822 20164920 PMC2826709

[B3] Butler K. Banday A. R. (2023). APOBEC3-mediated mutagenesis in cancer: causes, clinical significance and therapeutic potential. J. Hematol. Oncol. 16 (1), 31. 10.1186/s13045-023-01425-5 36978147 PMC10044795

[B4] Carr A. M. Lambert S. (2013). Replication stress-induced genome instability: the dark side of replication maintenance by homologous recombination. J. Mol. Biol. 425 (23), 4733–4744. 10.1016/j.jmb.2013.04.023 23643490

[B5] Carrai M. Campa D. Vodicka P. Flamini R. Martelli I. Slyskova J. (2017). Association between taste receptor (TAS) genes and the perception of wine characteristics. Sci. Rep. 7 (1), 9239–9247. 10.1038/s41598-017-08946-3 28835712 PMC5569080

[B6] Carter S. L. Eklund A. C. Kohane I. S. Harris L. N. Szallasi Z. (2006). A signature of chromosomal instability inferred from gene expression profiles predicts clinical outcome in multiple human cancers. Nat. Genet. 38 (9), 1043–1048. 10.1038/ng1861 16921376

[B7] Chen C. Chen Y. Jin X. Ding Y. Jiang J. Wang H. (2022). Identification of tumor mutation burden, microsatellite instability, and somatic copy number alteration derived nine gene signatures to predict clinical outcomes in STAD. Front. Mol. Biosci. 9, 793403. 10.3389/fmolb.2022.793403 35480879 PMC9037630

[B8] Dendrou C. A. Petersen J. Rossjohn J. Fugger L. (2018). HLA variation and disease. Nat. Rev. Immunol. 18 (5), 325–339. 10.1038/nri.2017.143 29292391

[B9] Edholm E. S. Wilson M. Bengten E. (2011). Immunoglobulin light (IgL) chains in ectothermic vertebrates. Dev. Comp. Immunol. 35 (9), 906–915. 10.1016/j.dci.2011.01.012 21256861

[B10] Fu M. Huang Y. Peng X. Li X. Luo N. Zhu W. (2022). Development of tumor mutation burden-related prognostic model and novel biomarker identification in stomach adenocarcinoma. Front. Cell Dev. Biol. 10, 790920. 10.3389/fcell.2022.790920 35399509 PMC8983817

[B11] Girirajan S. Dennis M. Y. Baker C. Malig M. Coe B. P. Campbell C. D. (2013). Refinement and discovery of new hotspots of copy-number variation associated with autism spectrum disorder. Am. J. Hum. Genet. 92 (2), 221–237. 10.1016/j.ajhg.2012.12.016 23375656 PMC3567267

[B12] Guo J. Huang J. Zhou Y. Zhou Y. Yu L. Li H. (2018). Germline and somatic variations influence the somatic mutational signatures of esophageal squamous cell carcinomas in a Chinese population. BMC Genomics 19 (1), 538. 10.1186/s12864-018-4906-4 30012096 PMC6048762

[B13] Guo J. Zhou Y. Xu C. Chen Q. Sztupinszki Z. Borcsok J. (2021). Genetic determinants of somatic selection of mutational processes in 3,566 human cancers. Cancer Res. 81 (16), 4205–4217. 10.1158/0008-5472.CAN-21-0086 34215622 PMC9662923

[B14] Gupta S. Won H. Chadalavada K. Nanjangud G. J. Chen Y. B. Al-Ahmadie H. A. (2022). TERT copy number alterations, promoter mutations and rearrangements in adrenocortical carcinomas. Endocr. Pathol. 33 (2), 304–314. 10.1007/s12022-021-09691-0 34549366 PMC9135779

[B15] Hastings P. J. Lupski J. R. Rosenberg S. M. Ira G. (2009). Mechanisms of change in gene copy number. Nat. Rev. Genet. 10 (8), 551–564. 10.1038/nrg2593 19597530 PMC2864001

[B16] Hellmann M. D. Callahan M. K. Awad M. M. Calvo E. Ascierto P. A. Atmaca A. (2018). Tumor mutational burden and efficacy of nivolumab monotherapy and in combination with ipilimumab in small-cell lung cancer. Cancer Cell 33 (5), 853–861.e4. 10.1016/j.ccell.2018.04.001 29731394 PMC6750707

[B17] Hovhannisyan G. Harutyunyan T. Aroutiounian R. Liehr T. (2019). DNA copy number variations as markers of mutagenic impact. Int. J. Mol. Sci. 20 (19), 4723. 10.3390/ijms20194723 31554154 PMC6801639

[B18] Jackson K. J. L. Wang Y. Gaeta B. A. Pomat W. Siba P. Rimmer J. (2012). Divergent human populations show extensive shared IGK rearrangements in peripheral blood B cells. Immunogenetics 64 (1), 3–14. 10.1007/s00251-011-0559-z 21789596

[B19] Jamal-Hanjani M. Wilson G. A. McGranahan N. Birkbak N. J. Watkins T. B. K. Veeriah S. (2017). Tracking the evolution of non-small-cell lung cancer. N. Engl. J. Med. 376 (22), 2109–2121. 10.1056/NEJMoa1616288 28445112

[B20] Keller A. Zhuang H. Chi Q. Vosshall L. B. Matsunami H. (2007). Genetic variation in a human odorant receptor alters odour perception. Nature 449 (7161), 468–472. 10.1038/nature06162 17873857

[B21] Long J. Delahanty R. J. Li G. Gao Y. T. Lu W. Cai Q. (2013). A common deletion in the APOBEC3 genes and breast cancer risk. J. Natl. Cancer Inst. 105 (8), 573–579. 10.1093/jnci/djt018 23411593 PMC3627644

[B22] Macé A. Kutalik Z. Valsesia A. (2018). Copy number variation. Methods Mol. Biol. 1793, 231–258. 10.1007/978-1-4939-7868-7_14 29876900

[B23] McCarroll S. A. Kuruvilla F. G. Korn J. M. Cawley S. Nemesh J. Wysoker A. (2008). Integrated detection and population-genetic analysis of SNPs and copy number variation. Nat. Genet. 40 (10), 1166–1174. 10.1038/ng.238 18776908

[B24] Raudvere U. Kolberg L. Kuzmin I. Arak T. Adler P. Peterson H. (2019). g:Profiler: a web server for functional enrichment analysis and conversions of gene lists (2019 update). Nucleic acids Res. 47 (W1), W191–W198. 10.1093/nar/gkz369 31066453 PMC6602461

[B25] Rizvi N. A. Hellmann M. D. Snyder A. Kvistborg P. Makarov V. Havel J. J. (2015). Cancer immunology. Mutational landscape determines sensitivity to PD-1 blockade in non-small cell lung cancer. Science 348 (6230), 124–128. 10.1126/science.aaa1348 25765070 PMC4993154

[B26] Shi K. Carpenter M. A. Banerjee S. Shaban N. M. Kurahashi K. Salamango D. J. (2017). Structural basis for targeted DNA cytosine deamination and mutagenesis by APOBEC3A and APOBEC3B. Nat. Struct. Mol. Biol. 24 (2), 131–139. 10.1038/nsmb.3344 27991903 PMC5296220

[B27] Stavnezer J. Schrader C. E. (2014). IgH chain class switch recombination: mechanism and regulation. J. Immunol. 193 (11), 5370–5378. 10.4049/jimmunol.1401849 25411432 PMC4447316

[B28] Steele C. D. Abbasi A. Islam S. M. A. Bowes A. L. Khandekar A. Haase K. (2022). Signatures of copy number alterations in human cancer. Nature 606 (7916), 984–991. 10.1038/s41586-022-04738-6 35705804 PMC9242861

[B29] van Opheusden B. Acerbi L. Ma W. J. (2020). Unbiased and efficient log-likelihood estimation with inverse binomial sampling. PLoS Comput. Biol. 16 (12), e1008483. 10.1371/journal.pcbi.1008483 33362195 PMC7758077

[B30] Watkins T. B. K. Lim E. L. Petkovic M. Elizalde S. Birkbak N. J. Wilson G. A. (2020). Pervasive chromosomal instability and karyotype order in tumour evolution. Nature 587 (7832), 126–132. 10.1038/s41586-020-2698-6 32879494 PMC7611706

[B31] Yicheng F. Xin L. Tian Y. Huilin L. (2022). Association of FLG mutation with tumor mutation load and clinical outcomes in patients with gastric cancer. Front. Genet. 13, 808542. 10.3389/fgene.2022.808542 36046250 PMC9421250

[B32] Zhou L. Huang W. Yu H-F. Feng Y-J. Teng X. (2020). Exploring TCGA database for identification of potential prognostic genes in stomach adenocarcinoma. Cancer Cell Int. 20 (1), 264. 10.1186/s12935-020-01351-3 32581654 PMC7310509

[B33] Zhu Y. H. Jeong S. Wu M. Jin Z. Y. Zhou J. Y. Han R. Q. (2019). Dietary intake of fatty acids, total cholesterol, and stomach cancer in a Chinese population. Nutrients 11 (8), 1730. 10.3390/nu11081730 31357492 PMC6723637

